# Methylation quantitative trait loci (meQTLs) are consistently detected across ancestry, developmental stage, and tissue type

**DOI:** 10.1186/1471-2164-15-145

**Published:** 2014-02-21

**Authors:** Alicia K Smith, Varun Kilaru, Mehmet Kocak, Lynn M Almli, Kristina B Mercer, Kerry J Ressler, Frances A Tylavsky, Karen N Conneely

**Affiliations:** 1 Department of Psychiatry and Behavioral Science, Emory University, 101 Woodruff Circle NE; Ste 4000, Atlanta, GA 30322, USA; 2 Genetics and Molecular Biology Program, Emory University, Atlanta, GA, USA; 3 Department of Preventive Medicine, University of Tennessee Health Science Center, Memphis, TN, USA; 4 Howard Hughes Medical Institute, Chevy Chase, MD, USA; 5 Department of Human Genetics, Emory University, Atlanta, GA, USA

**Keywords:** DNA methylation, meQTL, mQTL, Developmental stage, Ancestry, Race, Gene regulation, Inter-individual variation, Biomarker, Brain

## Abstract

**Background:**

Individual genotypes at specific loci can result in different patterns of DNA methylation. These methylation quantitative trait loci (meQTLs) influence methylation across extended genomic regions and may underlie direct SNP associations or gene-environment interactions. We hypothesized that the detection of meQTLs varies with ancestral population, developmental stage, and tissue type. We explored this by analyzing seven datasets that varied by ancestry (African American vs. Caucasian), developmental stage (neonate vs. adult), and tissue type (blood vs. four regions of postmortem brain) with genome-wide DNA methylation and SNP data. We tested for meQTLs by constructing linear regression models of methylation levels at each CpG site on SNP genotypes within 50 kb under an additive model controlling for multiple tests.

**Results:**

Most meQTLs mapped to intronic regions, although a limited number appeared to occur in synonymous or nonsynonymous coding SNPs. We saw significant overlap of meQTLs between ancestral groups, developmental stages, and tissue types, with the highest rates of overlap within the four brain regions. Compared with a random group of SNPs with comparable frequencies, meQTLs were more likely to be 1) represented among the most associated SNPs in the WTCCC bipolar disorder results and 2) located in microRNA binding sites.

**Conclusions:**

These data give us insight into how SNPs impact gene regulation and support the notion that peripheral blood may be a reliable correlate of physiological processes in other tissues.

## Background

DNA methylation patterns vary widely across the genome by developmental stage and by tissue type [1], making study design a challenge when the most relevant tissue is not accessible from living humans. This is particularly true for studies of neurologic or psychiatric traits. Although the most relevant tissues, like brain, may be obtained from non-human subjects, animal models often fail to adequately reflect the range of symptoms seen in humans. As a result, peripheral tissues are commonly used to identify biomarkers due to their ready availability.

A recent report characterized methylation patterns extensively in DNA derived from brain and blood samples taken from the same individuals [2]. The study confirmed that genome-wide DNA methylation is highly tissue-specific; however, the authors also noted a striking similarity between DNA methylation differences in the blood and brain (cerebellum and cortex) from the same individual, prompting the hypothesis that between-individual DNA methylation differences may be mediated by DNA sequence variation, which does not vary across tissues. Nevertheless, the study had only a limited number of subjects and was not designed to address this hypothesis directly.

Over the past few years, multiple studies [3-9] have revealed that DNA methylation at specific loci can be influenced by sequence variations, such that individual genotypes at a given locus may result in different patterns of DNA methylation due to allele-specific methylation. These sites are called methylation quantitative trait loci (meQTLs) and can influence the methylation pattern across an extended genomic region [3,8]. Numerous studies have found meQTLs in blood leukocytes, buccal cells, brain tissue, and lymphoblast cell lines [3-7], though there is only modest overlap between the SNP-CpG relationships identified in each study.

The ability to detect an meQTL is influenced by statistical power, which may depend on SNP allele frequency and linkage disequilibrium (LD) structure, which vary based on ancestry, or the variance of methylation at a specific CpG site, which may differ across stages of development or tissue type. These SNP-CpG relationships have wide-reaching biological relevance and likely contribute to inter-individual variation in gene regulation. The relationship between sequence variants and DNA methylation patterns in different sets of individuals may also provide insight into complex traits in which a particular environmental exposure associates with a trait only if incurred at a particular developmental window. Delineating sets of potential regulatory SNPs across different ancestries, developmental stages, and tissue types will help us assess the extent to which meQTLs can be used for large-scale studies of a wide range of human traits. This study marks an initial step in that process.

## Results

### meQTL detection

**Figure 1:**
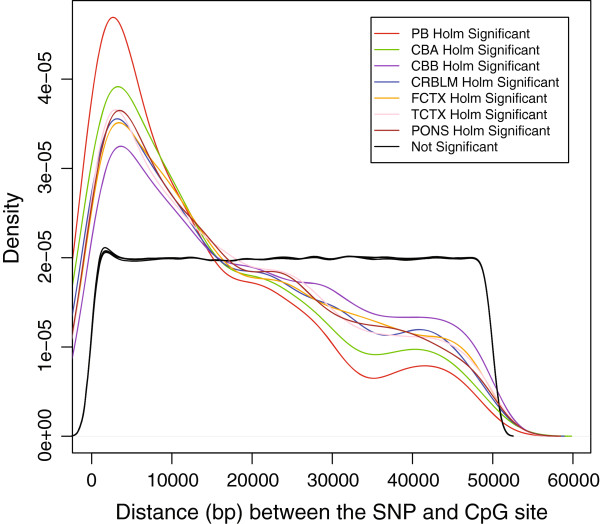
**Distribution (probability density function) of associated (colored) and unassociated (black) SNP-CpG pairs by absolute distance in each cohort.** Plot indicates that while the distance is roughly uniformly distributed between 0–50 kb for most pairs of SNPs and CpGs compared (black), the distance tends to be shorter (< 10 kb) for pairs where a meQTL was identified. Abbreviations: CB B: cord blood B, TCTX: temporal cortex, FCTX: frontal cortex, PONS: pons, CRBLM: cerebellum, CB A: cord blood A, and PB: peripheral blood.

We assessed genome-wide genotypes and DNA methylation in seven cohorts (described in Methods) 222,888 SNPs and 20,093 CpG sites that passed quality control (including a restriction of MAF > .05 for SNPs) in all seven cohorts were eligible for analysis. We tested a total of 529,224 unique SNP-CpG combinations where the SNP was within 50 kb of the CpG site. Numerous meQTLs were identified in each cohort after a conservative Holm adjustment for multiple testing (Additional file 1). To make comparisons between meQTL detection, we limited to only the SNP-CpG combinations that were present in every cohort (Table 1). On average, the distance between associated SNPs and CpG sites was ~15 kb (Figure 1) and did not differ substantially between cohorts. In each of the cohorts, the majority of meQTLs mapped to intronic regions (49.2-50.1%), though a limited number appeared to occur in synonymous (1.7-1.9%) or nonsynonymous coding (1.5-1.7%) SNPs. There was substantial overlap between the set of SNPs identified as meQTLs in this study and meQTLs (86.6-90.1%) or eQTLs (1.6-5.2%) identified in previous reports [8,10]. We used an empirical sampling strategy (described in Methods) to examine whether this overlap was significant: in 10,000 randomly chosen sets of SNP with similar MAF, we found 0 instances of overlap as great as the overlap in the original data, indicating that the observed overlap is significantly greater than expected by chance (p < 1×10^-4^).

**Table 1:** Number of meQTLs detected in each dataset and the number and percent overlapping between datasets, in the form number (percent)

	**# of**	**PB**	**CB A**	**CB B**	**FCTX**	**TCTX**	**CRBLM**	**PONS**
**Cohort**	**meQTLs**	**724**	**629**	**2055**	**2853**	**3029**	**2802**	**2150**
**PB**	**724**	-	319 (50.7)	366 (17.8)	254 (8.9)	234 (7.7)	185 (6.6)	203 (9.4)
**CB A**	**629**	319 (44.1)	-	437 (21.3)	205 (7.2)	196 (6.5)	165 (5.9)	211 (9.8)
**CB B**	**2055**	366 (50.6)	437 (69.5)	-	650 (22.8)	649 (21.4)	517 (18.5)	587 (27.3)
**FCTX**	**2853**	254 (35.1)	205 (32.6)	650 (31.6)	-	2046 (67.6)	1078 (38.5)	1414 (65.8)
**TCTX**	**3029**	234 (33.3)	196 (31.2)	649 (31.6)	2046 (71.7)	-	1091 (38.9)	1480 (68.9)
**CRBLM**	**2802**	185 (25.6)	165 (26.2)	517 (25.2)	1078 (37.8)	1091 (36.0)	-	1004 (46.7)
**PONS**	**2150**	203 (28.0)	211 (33.6)	587 (28.6)	1414 (49.6)	1480 (48.9)	1004 (35.8)	-

Bold numbers are the total meQTLs identified in each cohort and serve as the denominator for calculation of percentages.

Upper triangle: number (percent) of meQTLs identified in top dataset (column header) that were also identified in left-hand dataset (row header).

Lower triangle: number (percent) of meQTLs identified in left-hand dataset (row header) that were also identified in top dataset (column header).

*Abbreviations*: *CB B* cord blood B, *TCTX* temporal cortex, *FCTX* frontal cortex, *PONS* pons, *CRBLM* cerebellum, *CB A* cord blood A, and *PB* peripheral blood.

### Comparison of meQTLs by cohort

Of the meQTLs identified in each cohort, 10.6-49.5% were unique to that cohort. Note that the number of meQTLs identified varied substantially across cohorts, in part due to differences in power across cohorts. For example, 2055 meQTLs were found in umbilical cord blood from Caucasian neonates (CB B) compared to 629 in umbilical cord blood from African American neonates (CB A). Although these two groups had the same sample size, linkage disequilibrium between markers is substantially lower in African Americans than in Caucasians [11], so SNPs in CB A are less likely to tag untyped meQTLs. Despite the between-cohort differences in power, a number of SNP-CpG associations were common across cohorts. To determine whether the observed overlap between any two cohorts was significantly different from that expected by chance, we used Fisher’s exact test as described in Methods. There was significant overlap in meQTL detection between CB A and CB B (Table 1; overlap of 21.3-69.5%, depending on which group was treated as the baseline). Further, comparisons of meQTLs detected in African American samples at birth (CB A) and adult peripheral blood (PB) had similar agreement (44.1-50.7%). Finally, comparisons between Caucasian blood samples (CB B) and brain tissues (FCTX, TCTX, CRBLM, and PONS) also demonstrated greater overlap in detected meQTLs than would be expected by chance (18.5-31.6%). However, the highest proportions of overlap occurred between brain regions (35.8-71.7%). There is 6.6-35.1% overlap between PB and the four brain regions, even though the cohorts differ by both ancestral group and tissue type. All of the overlap between pairs of cohorts mentioned above was highly significant (Fisher’s exact p < 1×10^-308^); however, a caveat of this analysis is that the four brain regions were sampled from the same set of individuals. To investigate whether the higher levels of overlap between brain tissues are due to the sampling of brain tissue from a common set of subjects, we also calculated overlap from pairs of brain samples after randomly partitioning the subjects into two non-overlapping groups. Additional file 2 shows that for each pairwise comparison, the overlap between detected meQTLs is only slightly smaller in cross-tissue comparisons when comparing tissue sampled from non-overlapping groups (i.e., group A vs. B) vs. sampled from the same group (A vs. A, or B. vs. B). For A vs. B comparisons, we again observed a significantly larger number of overlapping meQTLs than expected by chance (Fisher exact p-values < 10^-284^ for all comparisons) despite having much lower power in these partitioned analyses, suggesting that the observed overlap between brain tissues is not an artifact caused by the sampling of brain tissue from a common set of subjects.

To verify that the overlap observed in Table 1 is robust to differences in linkage disequilibrium (LD) patterns among SNPs, we also performed secondary analyses where we examined only a set of roughly independent SNPs. Although fewer meQTLs were detected overall because fewer comparisons were made, we observed similarly large proportions of overlap in meQTL detection across cohorts when working with this pruned set of SNPs (Additional file 3). To verify that our observed associations are robust to how methylation was modeled, we re-performed select analyses where we modeled methylation using M-values (the logit transform of β-values [12]) rather than modeling β-values directly. Resulting test statistics for β-values vs. M-values were extremely similar; a representative example is shown in Additional file 4.

**Figure 2:**
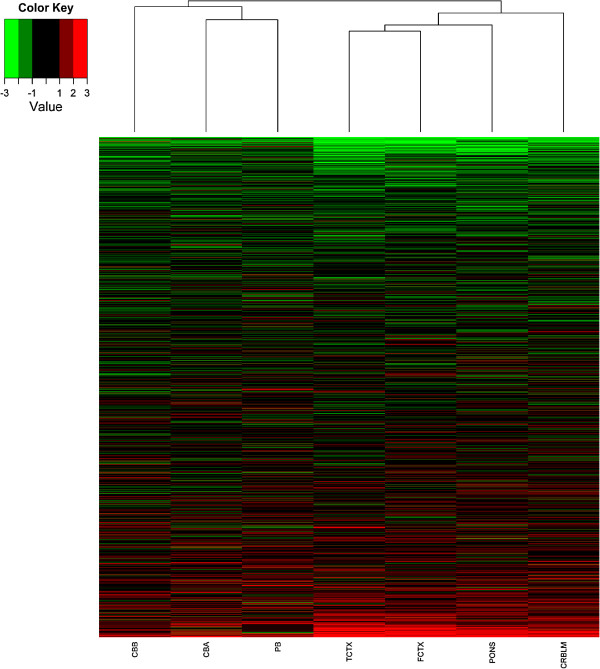
**Hierarchical clustering heatmap showing similarities of t-statistics of the meQTLs across all cohorts.** Each row represents one SNP-CpG site tested; only SNP-CpG combinations that were significant in at least one cohort are included here. Columns represent cohorts (labels at bottom), and the hierarchical clustering tree shows relative similarity in test statistics between the tissues and cohorts. Color represents strength and direction of association t-statistics (see color key). Cohort abbreviations from left to right: CB B: cord blood B, TCTX: temporal cortex, FCTX: frontal cortex, PONS: pons, CRBLM: cerebellum, CB A: cord blood A, and PB: peripheral blood.

To visually evaluate the similarities and differences between cohorts, a hierarchical clustering analysis was performed on the t-statistics from the meQTL association tests (Figure 2). Only the 7323 SNP-CpG combinations that were Holm-significant in at least one cohort were included in this analysis. The heatmap in Figure 2 shows that many of the meQTLs were consistent across the seven cohorts (top and bottom of heatmap), while others varied considerably between tissues (middle of heatmap). Among these SNP-CpG combinations, 34.5% had t-statistics with the same sign (i.e., associations in the same direction) for all seven cohorts, compared to the 1.67% expected by chance (computed as ∏ *p*_*i*_ + ∏ (1 - *p*_*i*_), where *p*_*i*_ represents the proportion of positive t-statistics in cohort *i* and ranges from .456 to .493 in our seven cohorts). Similarly, 62.0% of SNP-CpG combinations had the same sign for at least six of the seven cohorts, compared to 13.0% expected by chance. Although in many cases the associations were non-significant, this pattern is consistent with variable power to detect these meQTLs across cohorts. The hierarchical clustering tree in Figure 2 shows that the cohorts cluster primarily by ancestral group, likely reflecting differential power due to allele frequency differences between African American and Caucasian populations. The tree again demonstrates that the pattern of meQTL direction and significance is most similar between the different brain regions, particularly FCTX and TCTX. However, there are many similarities across tissue types. For example, in the comparisons between cerebellum (CRBLM) and cord blood (CB B), 77.2% showed test statistics in the same direction, as opposed to the 50.2% expected by chance. All of the above differences were highly significant according to binomial tests (p < 10^-200^).

**Figure 3:**
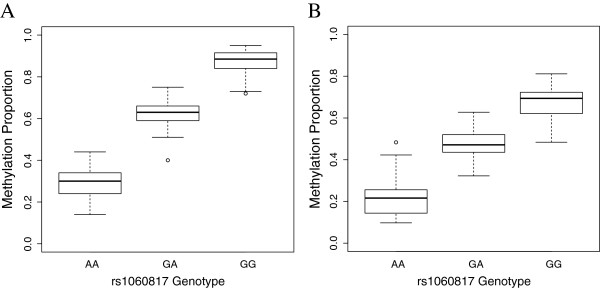
**Identification of meQTLs in multiple tissues.** rs10760117 associates with DNA methylation of cg21717724 in representative plots: CB B **(A)** and FCTX **(B)**. See Additional file 6 for the remaining cohorts and tissues.)

Sixty-seven SNP-CpG associations were identified as Holm-significant in all cohorts, independent of ancestry, age, or tissue type; these SNP-CpG pairs tended to be in or near 11 genes, many of which are involved in common biological processes, such as cell cycle progression (Additional file 5). For example, a SNP (rs10760117) approximately 35 base pairs upstream of the seventh exon of *PSMD5* associates with multiple CpG sites (cg09419670 and cg21717724) in the *PSMD5* promoter in all cohorts examined (8.47×10^-55^ < p < 1.14×10^-14^; Figure 3 and Additional file 6). Note that while a similar association between genotype and methylation is observed in all tissues, the range of methylation proportions varies considerably across tissues.

### Differences in meQTLs by tissue, developmental stage, and ancestral group

**Figure 4:**
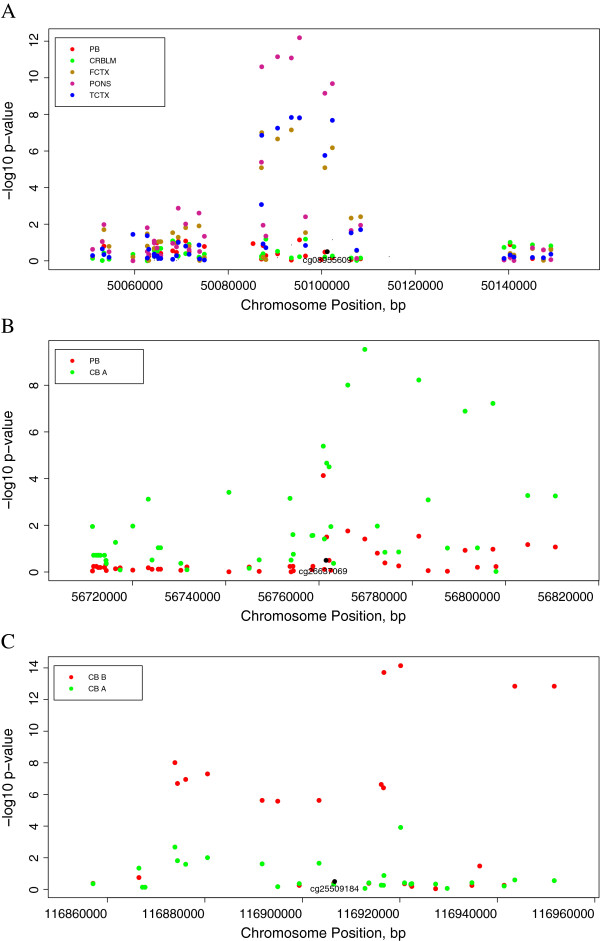
**Differences in meQTL detection between (A) tissue types; *APOE*, (B) developmental stage; *APLNR*, and (C) ancestry; *CFTR*.** Each plot displays meQTL associations for a single CpG site (labeled at bottom center of plot): the x-axis represents genomic position of SNPs, while the y-axis represents the -log p-value of the association between the SNP and the CpG site. Cohort abbreviations: PB: peripheral blood, CB A: cord blood A, CB B: cord blood B, CRBLM: cerebellum, TCTX: temporal cortex, FCTX: frontal cortex, and PONS: pons.

Examining the differences between these patterns may provide insight into SNP associations relevant to a specific tissue (Figure 4A), developmental stage (Figure 4B), or ancestral group (Figure 4C). Specifically, we found that genetic variation in and near *APOE* (apolipoprotein E) was associated with methylation patterns (cg14123992) in FCTX, TCTX, and PONS (minimum p = 6.50×10^-13^), but not even nominally associated in CRBLM or PB (Figure 4A, p > .05). Similarly, SNPs in or near *APLNR* (aka *AGTRL1*; apelin receptor) associated with methylation of a probe (cg26637069) in CB A (minimum p = 2.91×10^-10^), but not in adult PB (Figure 4B). Finally, SNPs near *CFTR* (cystic fibrosis transmembrane conductance regulator) demonstrated Holm-significant genotype-dependent methylation patterns (cg25509184) in CB B (minimum p = 7.26×10^-15^) but not CB A (all p > 1.21×10^-4^, Figure 4C). Interestingly, this cannot be attributed to differences in the minor allele frequencies between CB A (average MAF = 26.0%) and CB B (average MAF = 25.2%).

### GWAS enrichment

To compare our findings with earlier results demonstrating that meQTLs in cerebellum tissue were enriched in GWAS results for complex traits [9], we investigated whether our brain meQTLs were more likely to be represented among the top SNP associations from the WTCCC bipolar disorder study (defined here as p < 0.001, to be consistent with [9]). We found that the Holm-significant meQTLs identified in PONS were enriched (empirical p-value = .019) for SNP associations from the GWAS. When we performed a similar analysis using a looser definition of meQTLs (p < .001), we saw enrichment in FCTX (p < .0001), TCTX (p = .048), and PONS (p = .005), though not CRBLM, as observed in [9].

### miRNA enrichment

The meQTLs identified in this study were more likely to occur in miRNA binding sites (3.7-5.2%) than SNPs that were not meQTLs (2.9-3.1%) in multiple cohorts: FCTX (empirical p-value = .002), TCTX (p = .001), CRBLM (p < 1×10^-4^), PONS (p = .014), PB (p = .001), and CB A (p = .015). For example, rs3814309 associates with the proportion of DNA methylation at cg04987894 (*GSTM5*) in the FCTX (p = 1.22×10^-16^), TCTX (p = 2.68×10^-12^), CRBLM (p = 2.95×10^-11^), PONS (p = 9.88×10^-14^), CB A (7.76×10^-6^), CB B (p = 3.40×10^-4^), and PB (p = 2.63×10^-4^). Interestingly, rs3814309 also lies on a binding site for hsa-miR-1237 in all brain tissues examined.

## Discussion

This study compared meQTLs in seven cohorts of similar size and coverage. Although the number of meQTLs identified varied, those detected were often specific to a single cohort. Despite this, we saw a highly significant degree of overlap between cohorts made up of subjects from different developmental stages and ancestral groups, as well as between samples obtained from different tissues. Exploring the nature of the similarities and differences from this study will give us insight into the use of meQTLs for the interpretation of large-scale studies.

DNA methylation patterns vary substantially between different tissues [2], but the sequence variants influencing methylation patterns may be consistent across tissues, as exemplified in Figure 2 and Table 1. This study uncovered a correlation between meQTLs identified in umbilical cord blood and those identified in several brain regions in an independent set of adult samples. Some of these meQTLs are located in genes with common functions in the blood and brain. For example, in CB B and each of the brain cohorts, we saw multiple meQTLs across a region of up to 20.3-kb that associated with a CpG site near ATP-binding cassette, sub-family C (*ABCC4*). This gene encodes a drug transporter that functions at the blood–brain barrier [13]. Still other genes have functions that are exclusive in the brain. For example, in CB B and each of the brain cohorts, meQTLs associated with a CpG site near 5-hydroxytryptamine (serotonin) receptor 6 (*HTR6*; Additional file 1), which is expressed on in many brain regions and plays a role in cognition [14,15]. Recently, Lambe and colleagues characterized *HTR6* expression levels in postmortem tissue over time [16]. *HTR6* expression varies substantially over the lifespan suggesting that there may be particular ages at which one is more susceptible to psychiatric illnesses. Thus, examination of genotype-dependent DNA methylation of *HTR6* in peripheral blood may yield new information about the dynamic regulatory processes occurring in the brain. In contrast, genetic variation in and near *APOE* associates with methylation patterns of a CpG site in the first intron of that gene in FCTX, TCTX, and PONS, but not in CRBLM or PB. *APOE* is linked to Alzheimer’s disease [17], and this study suggests that there may be limited insight to be gained from studies of *APOE* regulation that are not conducted in specific brain regions.

When comparing meQTLs detected in the blood of neonates (CB A) versus an adult population (PB), we again noted substantial (44.1-50.7%) though not complete overlap. This is not particularly surprising since DNA methylation patterns differentiate by developmental stage as well as by tissue type [1]. Thus, if a gene involved in growth and development is less active in adulthood, meQTLs could be less detectable in adults based on the variability in DNA methylation levels of that gene. For example, in this study, we found associations between several SNPs and a CpG site in the promoter of the apelin receptor (*AGTRL1*) that were observable in blood from neonates but not adults. Apelin and its receptor help to stimulate hematopoiesis in embryonic stem cells, and dysregulation of these genes is linked to numerous disorders related to blood flow problems and hypertension [18-21]. Because fetal development is a period of extensive cellular replication and growth, epigenetic changes may establish long-term patterns among exposed individuals [22-24]. Extrapolating from this, genetic variation may be more influential during specific periods across the lifespan. Indeed, a recent study of meQTLs suggests that, in some cases, DNA methylation may be the mechanism by which genetic and environmental risk factors contribute to age-related phenotypes, including LDL, lung function, and longevity [25].

The power to detect meQTLs may be influenced by variations in allele frequencies and LD structure between cohorts, meaning we would expect to see differences in cohorts in which the length of expected LD blocks vary. Consistent with this, there were 3.3 times more meQTLs detected in the Caucasian cord blood cohort (CB B) compared to the African American cord blood cohort (CB A). We saw significant, albeit incomplete, overlap between meQTLs identified in both cohorts. In fact, 14.0-45.1% were specific to either CB A or CB B. For example, SNPs near *CFTR* (cystic fibrosis transmembrane conductance regulator) associate with methylation of a CpG site in the promoter region of *CFTR* in umbilical cord blood samples from Caucasian but not African American neonates. Genetic variants in *CFTR* cause cystic fibrosis, a Mendelian condition that is more prevalent in Caucasians [26,27]. Methylation-associated SNPs may be in LD with functional mutations that are more common in Caucasians than in African Americans, but the difference in genotype-dependent methylation patterns cannot be attributed to differences in the minor allele frequencies between the two cohorts. DNA methylation patterns vary significantly by ancestry [28-30], prompting several to suggest that epigenetic differences may contribute to the increased risk of African Americans for a variety of health conditions [31-33]. Studies of epigenetic differences may provide insight into health disparities [34], and meQTLs can help distinguish between genetic and epigenetic effects in such studies.

Because of their regulatory potential, identification of meQTLs may also be useful to harness the power of GWAS more efficiently [35]. The majority of meQTLs from this study localized to intronic regions. Despite this, we still saw significant enrichment of the meQTLs from FCTX, TCTX, and PONS in a GWAS study of bipolar disorder (BPD) [36]. A recent study suggests that the top associations from a GWAS of BPD are enriched for meQTLs in a cerebellum dataset; when only the SNPs that associated with DNA methylation patterns were examined for association with BPD, associations that met Bonferroni criteria were identified in multiple cohorts [9]. In addition, the results of this study suggest enrichment of miRNA binding sites among meQTLs, indicating these SNPs may promote a more complex degree of epigenetic regulation.

Though we attempted to present results from similarly powered cohorts, this study has several limitations. We would expect to identify more meQTLs if the cohorts were larger or if DNA methylation were assessed more densely. Due to study design and cohort availability, between-ancestry comparisons were limited to neonates. While DNA methylation was assessed in distinct tissues, those tissues contain multiple cell types. Thus, PB included the full range of leukocyte subpopulations, and the brain regions contained both a variety of neuronal phenotypes and glial cells. The corrections for multiple comparisons were conservative, and we have every confidence that the sets of meQTLs we identified are robust. We would expect, however, to have greater power to detect meQTLs in a study of homogenous cell types. In addition, bisulfite conversion does not distinguish between methylcytosine and hydroxymethylcytosine, which may be present in varying proportions in the seven cohorts used for this study.

## Conclusions

Our findings reveal similarities in genotype-dependent DNA methylation across a diverse range of subject characteristics and tissues. Though DNA methylation patterns can be highly tissue specific, the contribution of inter-individual variation in gene regulation remains understudied. We need to further explore and characterize such regions of the genome to facilitate population-based studies of disease and for biomarker development. More specifically, these data support the utility of studying peripheral blood when the ideal tissue is unavailable and provide scope for interpreting those results.

## Methods

### Cohorts

Adult peripheral blood (PB) was assessed in African American subjects recruited as part of a larger study investigating the influence of genetic and environmental factors on response to stressful life events in a predominantly urban population of low socioeconomic status [37,38]. Umbilical cord blood data were obtained from previous investigations in the CANDLE study, which assesses developmental outcomes in a community cohort from Shelby County, TN [39]. Because CANDLE includes African Americans and Caucasians in roughly equal proportions, the dataset was divided into two cohorts representing umbilical cord blood from African American (CB A) and Caucasian (CB B) neonates. Finally, publically available DNA methylation data were obtained for post-mortem samples of the frontal cortex (FCTX), temporal cortex (TCTX), cerebellum (CRBLM), and pons (PONS) of a single group of neurologically normal adult Caucasian subjects [8]. The characteristics of the seven cohorts examined in this study are summarized in Table 2. The institutional review boards for Emory University and Grady Memorial Hospital (for PB) as well as the University of Tennessee Health Science Center (for CB A and CB B) approved their respective studies. For the FCTX, TCTX, CRBLM, and PONS cohorts, individual-level data were obtained in accordance with an active Data Use Certification (to A.K.S.) for GEO Accession Number GSE15745 and dbGAP Study Accession phs000249.v1.p1.

**Table 2:** Characteristics of the study cohorts

**Dataset**	**Abbreviation**	**N**	**Ancestry**	**Age, yrs (mean ± SD)**	**Sex (% Male)**
Peripheral blood	PB	90	African American	42.5 ± 14.0	57.7
Cord blood A	CB A	87	African American	0.0 ± 0.0	55.2
Cord blood B	CB B	87	Caucasian	0.0 ± 0.0	44.8
Frontal cortex	FCTX	111	Caucasian	47.6 ± 23.8	67.6
Temporal cortex	TCTX	125	Caucasian	48.2 ± 24.5	66.9
Cerebellum	CRBLM	105	Caucasian	46.6 ± 23.0	71
Pons	PONS	106	Caucasian	46.5 ± 24.0	69.6

### DNA methylation data

The methylation datasets for this analysis have been described previously [8,30,40]. For each cohort, DNA methylation was first assessed at 27,578 CpG sites using the HumanMethylation27 BeadChip (Illumina). Samples with probe detection call rates < 90% were excluded, as were those with an average intensity value of either < 50% of the experiment-wide sample mean or < 2,000 arbitrary units. Data points with detection p-values > 0.001 were set to missing. Estimated DNA methylation proportions or β-values were then computed for each CpG site and sampled as the ratio of methylated signal to total (methylated + unmethylated) signal. Principal components analysis (PCA) of the β-values was then used to identify and eliminate 9 outliers from PONS, 21 from FCTX, and 6 from CRBLM, where outliers were determined as samples that fell more than 2.5 standard deviations away from the origin in plots of the first two principal components (Additional file 7). Additional file 7 also indicates batch effects in the brain samples, so we adjusted for these in our analysis as described below. No other cohort had evidence of outliers, and samples were hybridized in a single batch for each of the other cohorts. CpG sites with 1000 Genomes Project (Pilot 1 Data Release 2010_03) variants physically contained within the Illumina probe were excluded from all analyses, since CpG-SNP associations involving sites with variants in the probe are likely to be technical artifacts. After this step, 20,093 CpG sites passing QC in all cohorts remained eligible for analysis.

### Genotyping data

Genotyping for each cohort has been described previously [8,30]. Genotyping was performed using multiple platforms: Illumina Omni-Quad 1 M, Omni-Express BeadChips (PB), HumanHap550 (FCTX, TCTX, CRBLM, PONS), and Affymetrix Genome-Wide Human SNP Array 5.0 and 6.0 (CB A and CB B). PLINK was used to perform quality control analyses such that, for all datasets, SNPs that had a call rate < 95%, a minor allele frequency (MAF) < .05, or significant deviation from Hardy-Weinberg proportions (p < .00001) were excluded, as were samples with > 5% missing data. So that annotation information was consistent between all datasets, the positional information for all the genotype datasets was converted to build 36 of the Human genome using the UCSC liftover tool when necessary [41]. All allelic designations are oriented to the forward strand.

MaCH 1.0 [42] was used to impute missing genotypes for SNPs that were not common to each array. Caucasian datasets were imputed using the HapMap CEU + TSI phase 3 reference samples, and the African American datasets were imputed using unrelated individuals from HapMap ASW, CEU, LWK, MKK, TSI, and YRI Phase 3 reference samples. Imputed SNPs with an estimated r^2^ < .3 between imputed and true genotypes and those with posterior probabilities < .9 for the most likely genotype were excluded from subsequent analysis. After imputation, 222,888 SNPs with MAF > .05 passed quality control based on genotyping or imputation in all cohorts and were eligible for analysis.

### Statistical analysis

All statistical analyses were conducted in R. The relationship between the β-value or proportion of methylation at each CpG site and each SNP within 50 kb of that site was examined via linear regression, where β-values were modeled as a linear function of the number of reference alleles (0, 1, or 2). As appropriate, we included covariates to account for Affymetrix array 5.0 vs. 6.0 (CB A and CB B), or hybridization batch (FCTX, TCTX, CRBLM, PONS; other cohorts were hybridized in a single batch). To eliminate the possibility of inflated test statistics due to the lack of power at particular sites, analysis was restricted to SNP-CpG combinations with complete data for 32 or more subjects in each cohort (i.e., residual degrees of freedom ≥ 30). A Holm (step-down Bonferroni) correction [43] was applied to adjust for the total number of tests performed in each dataset; this type of approach will maintain the experiment-wide type I error rate at 5% for independent tests and will be even more conservative for the correlated tests performed here [44]. Gene ontology (GO) terms for biological processes were assigned using GeneCodis 2.0 [45,46]. We used SNAP to map the location of each meQTL relative to its location within a gene [47].

To test whether the overlap of meQTLs identified between each pair of cohorts was significantly greater than that expected by chance, we used Fisher’s exact test to test for independence between meQTL status in one cohort vs. meQTL status in another cohort. To test for enrichment in other datasets, we used an empirical sampling strategy. We first used this strategy to test for significant overlap between the set of meQTLs identified in each cohort and the set of variants listed in the Wellcome Trust GWAS for bipolar disorder [36]. To generate an empirical p-value for the enrichment test, for each cohort we randomly sampled sets of *X* SNPs from the full set of SNPs analyzed, where *X* is the number of SNPs identified as significant meQTLs in that cohort. To ensure that the distribution of MAF in the randomly drawn datasets was similar to the original, SNPs in each cohort were binned by MAF intervals of .025. For each random sample we then sampled the same number of SNPs from each bin as in the original set of significant meQTLs. For each random set, we computed the number of overlapping SNPs as the number of GWAS SNPs included in the random set of *X* SNPs, and compared this number to the number of overlapping SNPs in the original analysis. The empirical p-value was then the proportion of random SNP sets demonstrating at least as much overlap with the GWAS SNPs as observed in the original analysis.

We also used the strategy described above to compare enrichment of meQTLs from this study to published meQTL and eQTL results. For blood cohorts (PB, CB A, and CB B), we compared to eQTLs detected by Stranger and colleagues [10], and for brain cohorts (FCTX, TCTX, CRBLM, and PONS), we compared to meQTLs and eQTLs detected by Gibbs and colleagues [8]. Each enrichment test was conducted with 10,000 randomly sampled SNP sets, similar to those described above.

To identify meQTLs that could influence miRNA binding, we examined the list of SNPs in predicted miRNA-mRNA binding sites from the MirSNP database (http://cmbi.bjmu.edu.cn/mirsnp). Similarly, we tested for enrichment of the miRNA SNPs among the set of significant meQTLs and generated an empirical p-value using 1000 randomly sampled sets of SNPs for each cohort.

For secondary analyses involving an independent set of SNPs, we used PLINK to prune the genotype data in windows of 50 bp (base pairs), removing one SNP from each pair of SNPs with r^2^ > 0.05. We then performed select analyses on this reduced set of roughly independent SNPs (summarized in Additional file 3).

### Additional material

The additional material contains three figures and four tables that provide supporting information for specific points in the paper.

## Abbreviations

meQTL: Methylation quantitative trait loci; LD: Linkage disequilibrium (LD); CB A: Umbilical cord blood from African American neonates; CB B: Umbilical cord blood from Caucasian neonates; PB: Adult peripheral blood; FCTX: Frontal cortex; TCTX: Temporal cortex; CRBLM: Cerebellum; PONS: Pons; APOE: Apolipoprotein E; ABCC4: ATP-binding cassette, sub-family C; AGTRL1: Apelin receptor; CFTR: Cystic fibrosis transmembrane conductance regulator; HTR6: Serotonin receptor 6; BPD: Bipolar disorder; MAF: Minor allele frequency; GO: Gene ontology.

## Competing interests

The authors declare no competing interests.

## Authors’ contributions

AKS and KNC designed the experiments; LMA, KBM, KJR, and FAT performed the experiments or provided biological data; AKS, VK, MK, and KNC performed the analyses; AKS, VK, and KNC wrote the paper. All authors read and approved the final manuscript.

## Supplementary Material

Additional file 1: Table S1 meQTLs detected in each cohort. Click here for file

Additional file 2: Table S2 Number of meQTLs detected in each cohort and overlap between non-redundant samples. **Table S3.** Number of meQTLs detected and overlap between independent SNPs. **Table S4.** Gene ontology (GO) terms for meQTLs identified in all cohorts. Click here for file

Additional file 3: Figure S1 Identification of meQTLs in multiple tissues. rs10760117 associates with DNA methylation of cg21717724 in CB A (A), CB B (B), PB (C), FCTX (D), TCTX (E), CRBLM (F), and PONS (G). **Figure S2.** Principal component analysis used to identify and remove outliers from each cohort. **Figure S3.** T-statistics for Holm-significant CpG-SNP associations are extremely similar in analyses of β-values (Y-axis) vs. M-values (X-axis). Click here for file

Additional file 4 T-statistics for Holm-significant CpG-SNP associations are extremely similar in analyses of β-values (Y-axis) vs. M-values (X-axis). Click here for file

Additional file 5 Gene Ontology (GO) terms for mQTLs identified in all cohorts. Click here for file

Additional file 6 **Identification of meQTLs in multiple tissues.** rs10760117 associates with DNA methylation of cg21717724 in CB A (A), CB B (B), PB (C), FCTX (D), TCTX (E), CRBLM (F), and PONS (G). Click here for file

Additional file 7 Principal component analysis used to identify and remove outliers from each cohort. Click here for file
