# Supplementary material for: Methylation quantitative trait loci (meQTLs) are consistently detected across ancestry, developmental stage, and tissue type
Source: BMC Genomics. 2014 Feb 21;15:145. doi: 10.1186/1471-2164-15-145 (PMC4028873; doi:10.1186/1471-2164-15-145)
Supplement: Additional file 2: Table S2 — Number of meQTLs detected in each cohort and overlap between non-redundant samples. Table S3. Number of meQTLs detected and overlap between independent SNPs. Table S4. Gene ontology (GO) terms for meQTLs identified in all cohorts. [file 1471-2164-15-145-S2.DOCX]

Additional file 2: Number of meQTLs detected, and overlap, in all pairwise comparisons of the four brain tissues. For each comparison, we randomly partitioned all available samples into two non-overlapping subsets: group A and group B.

Format: # of meQTLs overlapping (fraction of meQTLs detected in column header, fraction of meQTLs in row header)

|  | FCTX |  |  |
| --- | --- | --- | --- |
| TCTX |  | Group A (1050 meQTL) | Group B (575 meQTL) |
|  | Group A (929 meQTL) | 488 (.46, .52) | 288 (.50, .31) |
|  | Group B (1013 meQTL) | 409 (.39, .40) | 366 (.36, .31) |

|  | PONS |  |  |
| --- | --- | --- | --- |
| TCTX |  | Group A (590 meQTL) | Group B (457 meQTL) |
|  | Group A (781 meQTL) | 285 (.48, .36) | 187 (.41, .24) |
|  | Group B (929 meQTL) | 295 (.50, .32) | 284 (.62, .31) |

|  | CRBLM |  |  |
| --- | --- | --- | --- |
| FCTX |  | Group A (636 meQTL) | Group B (536 meQTL) |
|  | Group A (362 meQTL) | 166 (.26, .46) | 139 (.26, .38) |
|  | Group B (450 meQTL) | 166 (.26, .36) | 164 (.31, .36) |

|  | CRBLM |  |  |
| --- | --- | --- | --- |
| PONS |  | Group A (606 meQTL) | Group B (585 meQTL) |
|  | Group A (373 meQTL) | 176 (.29, .47) | 145 (.24, .39) |
|  | Group B (304 meQTL) | 126 (.21, .41) | 124 (.21, .41) |

|  | CRBLM |  |  |
| --- | --- | --- | --- |
| TCTX |  | Group A (813 meQTL) | Group B (753 meQTL) |
|  | Group A (570 meQTL) | 211 (.26, .37) | 182 (.24, .32) |
|  | Group B (586 meQTL) | 183 (.23, .31) | 207 (.27, .35) |

|  | FCTX |  |  |
| --- | --- | --- | --- |
| PONS |  | Group A (504 meQTL) | Group B (588 meQTL) |
|  | Group A (437 meQTL) | 246 (.49, .56) | 222 (.38, .51) |
|  | Group B (418 meQTL) | 204 (.40, .49) | 266 (.45, .64) |
