# Supplementary material for: Methylation quantitative trait loci (meQTLs) are consistently detected across ancestry, developmental stage, and tissue type
Source: BMC Genomics. 2014 Feb 21;15:145. doi: 10.1186/1471-2164-15-145 (PMC4028873; doi:10.1186/1471-2164-15-145)
Supplement: Additional file 3: Figure S1 — Identification of meQTLs in multiple tissues. rs10760117 associates with DNA methylation of cg21717724 in CB A (A), CB B (B), PB (C), FCTX (D), TCTX (E), CRBLM (F), and PONS (G). Figure S2. Principal component analysis used to identify and remove outliers from each cohort. Figure S3. T-statistics for Holm-significant CpG-SNP associations are extremely similar in analyses of β-values (Y-axis) vs. M-values (X-axis). [file 1471-2164-15-145-S3.DOCX]

Additional file 3: Number of meQTLs detected in each dataset using only a set of 9713 LD-pruned SNPs and the number and percent overlapping between datasets, in the form # (%)

|  | **# of** | **PB** | **CB A** | **CB B** | **FCTX** | **TCTX** | **CRBLM** | **PONS** |
| --- | --- | --- | --- | --- | --- | --- | --- | --- |
| **Cohort** | **meQTLs** | **26** | **25** | **95** | **113** | **119** | **111** | **100** |
| **PB** | **26** |  | 8 (32.0) | 13 (13.7) | 12 (10.6) | 12 (10.1) | 9 (8.1) | 8 (8.0) |
| **CB A** | **25** | 8 (30.8) |  | 16 (16.8) | 10 (8.9) | 8 (6.7) | 7 (6.3) | 9 (9.0) |
| **CB B** | **95** | 13 (50.0) | 16 (64.0) |  | 32 (28.3) | 32 (26.9) | 27 (24.3) | 34 (34.0) |
| **FCTX** | **113** | 12 (46.2) | 10 (40.0) | 32 (33.7) |  | 73 (61.3) | 43 (38.7) | 53 (53.0) |
| **TCTX** | **119** | 12 (46.2) | 8 (32.0) | 32 (33.7) | 73 (64.6) |  | 42 (37.8) | 65 (65.0) |
| **CRBLM** | **111** | 9 (34.6) | 7 (28.0) | 27 (28.4) | 43 (38.1) | 42 (35.3) |  | 39 (39.0) |
| **PONS** | **100** | 8 (30.8) | 9 (36.0) | 34 (35.8) | 53 (46.9) | 65 (54.6) | 39 (35.1) |  |

Upper triangle: percent of meQTLs identified in top dataset (column header) that were also identified in left-hand dataset (row header).

Lower triangle: percent of meQTLs identified in left-hand dataset (row header) that were also identified in top dataset (column header).
