# Supplementary material for: Methylation quantitative trait loci (meQTLs) are consistently detected across ancestry, developmental stage, and tissue type
Source: BMC Genomics. 2014 Feb 21;15:145. doi: 10.1186/1471-2164-15-145 (PMC4028873; doi:10.1186/1471-2164-15-145)
Supplement: Additional file 4 — T-statistics for Holm-significant CpG-SNP associations are extremely similar in analyses of β-values (Y-axis) vs. M-values (X-axis). [file 1471-2164-15-145-S4.DOCX]

Additional file 4: T-statistics for Holm-significant CpG-SNP associations are extremely similar in analyses of β-values (Y-axis) vs. M-values (X-axis).
