# Supplementary material for: Methylation quantitative trait loci (meQTLs) are consistently detected across ancestry, developmental stage, and tissue type
Source: BMC Genomics. 2014 Feb 21;15:145. doi: 10.1186/1471-2164-15-145 (PMC4028873; doi:10.1186/1471-2164-15-145)
Supplement: Additional file 5 — Gene Ontology (GO) terms for mQTLs identified in all cohorts. [file 1471-2164-15-145-S5.DOCX]

Additional file 5: Gene Ontology (GO) terms for mQTLs identified in all cohorts.

| **Genes** | **GO Term** | **Biological Process** |
| --- | --- | --- |
| *C14orf58* |  |  |
| *C21orf128* |  |  |
| *HMOX2* | GO:0006778 | porphyrin-containing compound metabolic process |
|  | GO:0006788 | heme oxidation |
|  | GO:0006879 | cellular iron ion homeostasis |
|  | GO:0006979 | response to oxidative stress |
|  | GO:0042167 | heme catabolic process |
| *IRF6* | GO:0007050 | cell cycle arrest |
|  | GO:0030216 | keratinocyte differentiation |
|  | GO:0043588 | skin development |
|  | GO:0043616 | keratinocyte proliferation |
|  | GO:0048468 | cell development |
|  | GO:0060333 | interferon-gamma-mediated signaling pathway |
|  | GO:0060337 | type I interferon-mediated signaling pathway |
|  | GO:0060644 | mammary gland epithelial cell differentiation |
| *NUP214* | GO:0006406 | mRNA export from nucleus |
|  | GO:0006606 | protein import into nucleus |
|  | GO:0051726 | regulation of cell cycle |
| *PPIE* | GO:0000413 | protein peptidyl-prolyl isomerization |
| *PSMD5* | GO:0000075 | cell cycle checkpoint |
|  | GO:0000084 | S phase of mitotic cell cycle |
|  | GO:0000209 | protein polyubiquitination |
|  | GO:0000216 | M/G1 transition of mitotic cell cycle |
|  | GO:0002474 | antigen processing and presentation of peptide antigen via MHC class I |
|  | GO:0006521 | regulation of cellular amino acid metabolic process |
|  | GO:0006977 | DNA damage response, signal transduction by p53 class mediator resulting in cell cycle arrest |
|  | GO:0031145 | anaphase-promoting complex-dependent proteasomal ubiquitin-dependent protein catabolic process |
|  | GO:0051436 | negative regulation of ubiquitin-protein ligase activity involved in mitotic cell cycle |
|  | GO:0051437 | positive regulation of ubiquitin-protein ligase activity involved in mitotic cell cycle |
|  | GO:0051439 | regulation of ubiquitin-protein ligase activity involved in mitotic cell cycle |
|  | GO:0070682 | proteasome regulatory particle assembly |
| *RBFA (C18orf22)* | GO:0006364 | rRNA processing |
| *RPF2 (BXDC1)* |  |  |
| *UGT2B28* |  |  |
| *WDR45L* | GO:0006914 | autophagy |
|  | GO:0042594 | response to starvation |
