# Supplementary material for: Methylation quantitative trait loci (meQTLs) are consistently detected across ancestry, developmental stage, and tissue type
Source: BMC Genomics. 2014 Feb 21;15:145. doi: 10.1186/1471-2164-15-145 (PMC4028873; doi:10.1186/1471-2164-15-145)
Supplement: Additional file 6 — Identification of meQTLs in multiple tissues. rs10760117 associates with DNA methylation of cg21717724 in CB A (A), CB B (B), PB (C), FCTX (D), TCTX (E), CRBLM (F), and PONS (G). [file 1471-2164-15-145-S6.DOCX]

Additional file 6: Identification of meQTLs in multiple tissues. rs10760117 associates with DNA methylation of cg21717724 in CB A (A), CB B (B), PB (C), FCTX (D), TCTX (E), CRBLM (F), and PONS (G).

A

D

C

B

F

E

G
