# Supplementary material for: Methylation quantitative trait loci (meQTLs) are consistently detected across ancestry, developmental stage, and tissue type
Source: BMC Genomics. 2014 Feb 21;15:145. doi: 10.1186/1471-2164-15-145 (PMC4028873; doi:10.1186/1471-2164-15-145)
Supplement: Additional file 7 — Principal component analysis used to identify and remove outliers from each cohort. [file 1471-2164-15-145-S7.DOCX]

Additional file 7: Principal component analysis used to identify and remove outliers from each cohort.

**FCTX: before outlier removal and QC**

**FCTX: after outlier removal and QC**

**TCTX: before outlier removal and QC**

**TCTX: after outlier removal and QC**

**CRBLM: before outlier removal and QC**

**CRBLM: after outlier removal and QC**

**PONS: before outlier removal and QC**

**PONS: after outlier removal and QC**
